# Plasmonic Fano resonance and dip of Au-SiO_2_-Au nanomatryoshka

**DOI:** 10.1186/1556-276X-8-468

**Published:** 2013-11-08

**Authors:** Jiunn-Woei Liaw, Huang-Chih Chen, Mao-Kuen Kuo

**Affiliations:** 1 Department of Mechanical Engineering, Chang Gung University, 259 Wen-Hwa 1st Rd., Kwei-Shan, Taoyuan 333, Taiwan; 2 Center for Biomedical Engineering, Chang Gung University, 259 Wen-Hwa 1st Rd., Kwei-Shan, Taoyuan 333, Taiwan; 3 Institute of Applied Mechanics, National Taiwan University, 1, Sec. 4, Roosevelt Rd., Taipei 106, Taiwan

**Keywords:** Nanomatryoshka, Au-SiO_2_-Au, Fano resonance, Fano dip, Fano factor, Dyadic Green’s function, Radiative power, Nonradiative power, Scattering efficiency, Absorption efficiency

## Abstract

This study theoretically investigates Fano resonances and dips of an Au-SiO_2_-Au nanomatryoshka that is excited by a nearby electric dipole. An analytical solution of dyadic Green's functions is used to analyze the radiative and nonradiative power spectra of a radial dipole in the proximity of a nanomatryoshka. From these spectra, the plasmon modes and Fano resonances that accompany the Fano dips are identified. In addition, the scattering and absorption spectra of a nanomatryoshka that is illuminated by a plane wave are investigated to confirm these modes and Fano dips. Our results reveal that a Fano dip splits each of the dipole and quadrupole modes into bonding and anti-bonding modes. The Fano dip and resonance result from the destructive interference of the plasmon modes of the Au shell and the Au core. The Fano factors that are obtained from the nonradiative power spectra of the Au shell and the Au core of a nanomatryoshka are in accordance with those obtained from the absorption cross section spectra. Moreover, these Fano factors increase as the plasmonic coupling of the Au shell with the core increases for both dipole and quadrupole modes.

## Background

The interaction of an emitter with a nearby plasmonic nanostructure is an important topic in nanophotonics and nanooptics [1-7]. The effects of the surface-enhanced fluorescence of a plasmonic nanostructure on the photoluminescence of a molecule or quantum dot in its proximity have recently become more important [5-9]. Owing to the localized surface plasmon resonances (LSPR), the photoluminescence of an emitter can be modified - either enhanced or quenched [6]. More recently, the Fano resonance and dip of the external interference of two or more coupled plasmonic nanostructures, such as a dimer of two nanorods, have been studied [10-16]. Luk'yanchuk et al. provided a detailed review of Fano resonance, particularly that associated with external interference [17]. In the past decade, various plasmonic nanocomposites have been synthesized and proposed to exhibit Fano resonance, such as the Au-SiO_2_-Au nanomatryoshka [18-21]. In addition, the symmetry breaking of a nanomatryoshka due to the offset of the core from the shell can induce significant Fano resonance [19]. This paper studies the Fano resonance and dip of the internal interference in a nanomatryoshka, which is the electromagnetic (EM) coupling between Au shell and Au core. In particular, the effects of the Fano resonance and dip on the dipole and quadrupole modes are discussed. The Fano resonances and dips of an Au-SiO_2_-Au nanomatryoshka that are induced by a nearby dipole or an incident plane wave are investigated theoretically. The former phenomenon is analyzed using the dyadic Green's function in terms of spherical harmonic wave functions [22], and the latter is analyzed using the Mie theory [6]. The plasmon modes of this multi-layered structure are discussed. The Fano factors of the Au core and the Au shell of a nanomatryoshka that are obtained from the nonradiative power spectrum of an electric dipole and the absorption spectrum of a plane wave are analyzed and quantitatively compared. We have calculated the responses of a tangential dipole as well as a radial dipole interacting with the Ag nanoshell [23]. Both results at these plasmon modes are in accordance. However, the features of the plasmon modes of nanoshell excited by the radial dipole are more pronounced than those by the tangential dipole. Therefore, we are only dedicated to studying the responses of a radial dipole interacting with nanomatryoshka in this paper.

## Methods

**Figure 1:**
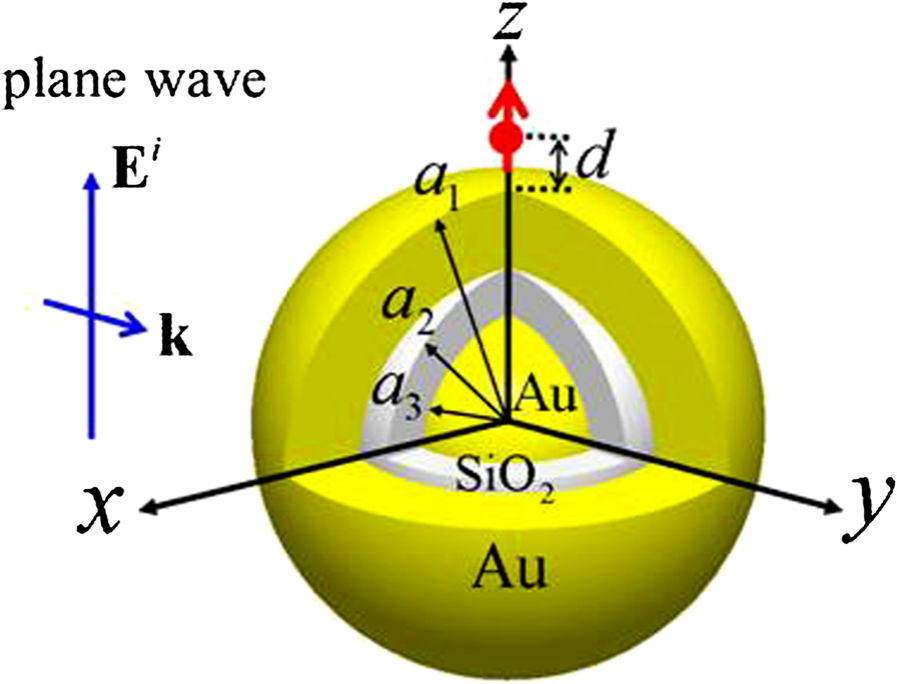
**Configuration of Au-SiO**_**2**_**-Au nanomatryushka irradiated by a radial electric dipole or a *z*-polarized plane wave.** The radii of the outer Au shell, the SiO_2_ shell, and the Au core are denoted by *a*_1_, *a*_2_, and *a*_3_, respectively.

Figure 1 shows the configuration of the Au-SiO_2_-Au nanomatryoshka, which consists of an SiO_2_ layer between an Au core and an Au shell, excited by a radial electric dipole or illuminated by polarized light. The outer radius of the Au shell, the radius of the middle silica layer, and the radius of the Au core are denoted by *a*_1_, *a*_2_, and *a*_3_, respectively. The thicknesses of the outer Au shell and the silica interlayer are denoted by *t*_1_ and *t*_2_, respectively, where *t*_1_ *= a*_1_ *- a*_2_, *t*_2_ *= a*_2_ *- a*_3_. Without loss of generality, the radial dipole is a distance *d* above the north pole of the nanomatryoshka, and the incident plane wave is assumed to propagate along the *y*-axis with a *z*-polarized electric field. The origin of the coordinate system is located at the center of the Au core. Throughout this paper, the classical theory of Maxwell's equations is used to analyze the electromagnetic field that is induced by an electric dipole or a plane wave that irradiates a nanomatryoshka. An analytical solution of the dyadic Green's functions is used in the former case [22], and the Mie theory is used in the latter case [23]. In response to the interaction of a radial dipole with the nearby nanomatryoshka, the radiative power can be expressed by

(1) Pr=12Re∫SE×H¯·da,

where the integral surface *S* can be any arbitrary closed surface that encloses the nanomatryoshka and the electric dipole [23]. The nonradiative power due to the ohmic loss in the nanomatryoshka is the dissipation power in metal,

(2) Pnr=-12Re∫SmE×H¯⋅da,

where *S*_m_ represents the outer surface of the Au shell [6,23]. Here, the unit normal is outward. Since the silica layer and its surrounding medium are lossless media, the nonradiative power is the total power dissipated in the Au shell and core, which can be decomposed into $P_{\mathrm{nr}}=P_{\mathrm{nr}}^{c}+P_{\mathrm{nr}}^{s}$. The dissipation power in the Au core is given by

(3) Pnrc=-12Re∫ScE×H¯⋅da,

where *S*_c_ is the surface of the Au core. The multi-connected surface of the Au shell is *S*_m_∪*S*_c_. Equations 2 and 3 can be used to analyze individually the contributions of the Au shell and the Au core.

Moreover, the Fano line-shape function in terms of wavelength *λ* is defined as

(4) $$F\left(\lambda \right)=\frac{A{\left(q+b\right)}^{2}}{\left(1+b^{2}\right)},$$

where $b=\left(1/\lambda -1/\lambda_{0}\right)/\left(\delta_{f}/\lambda_{0}^{2}\right)$[10-12]. In Equation 4, *q*, *λ*_0_, and *δ*_*f*_ are the Fano factor, the central wavelength, and the bandwidth, respectively. Here, *A* is a constant for amplitude. Below, this profile will be used to fit the spectra of the nonradiative powers or absorption efficiencies of the Au shell and the Au core at the Fano resonance.

## Results and discussion

**Figure 2:**
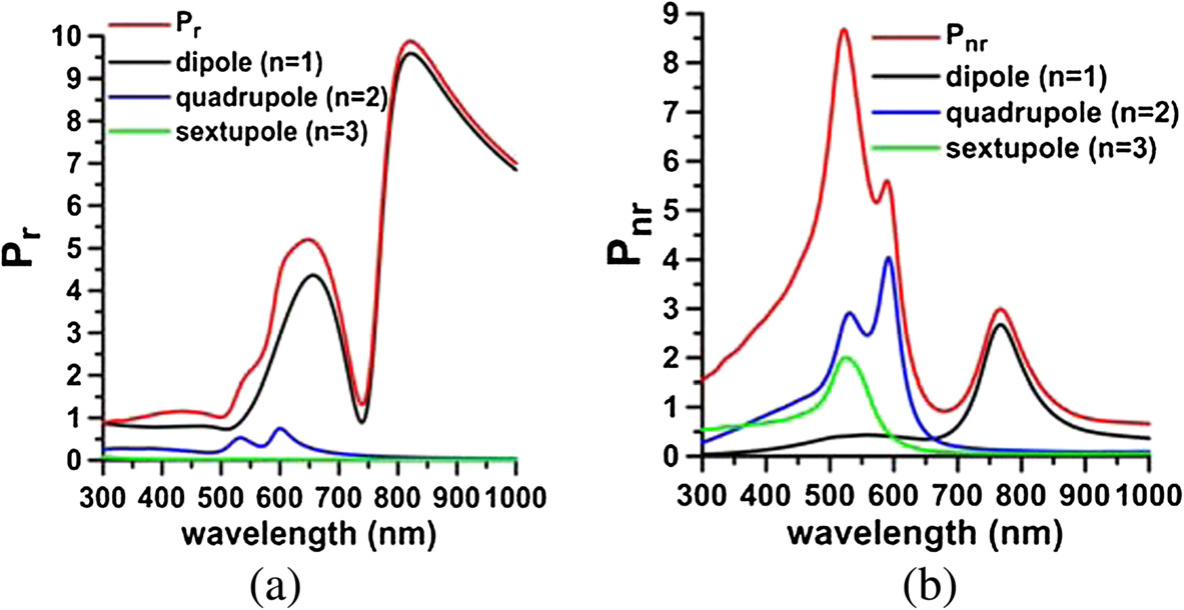
**Radiative powers (a) and nonradiative powers (b).** Component of each order mode of radial electric dipole interacting with a nanomatryushka of [*a*_1_*, a*_2_*, a*_3_] = [75, 50, 35] nm (*d* = 25 nm).

**Figure 3:**
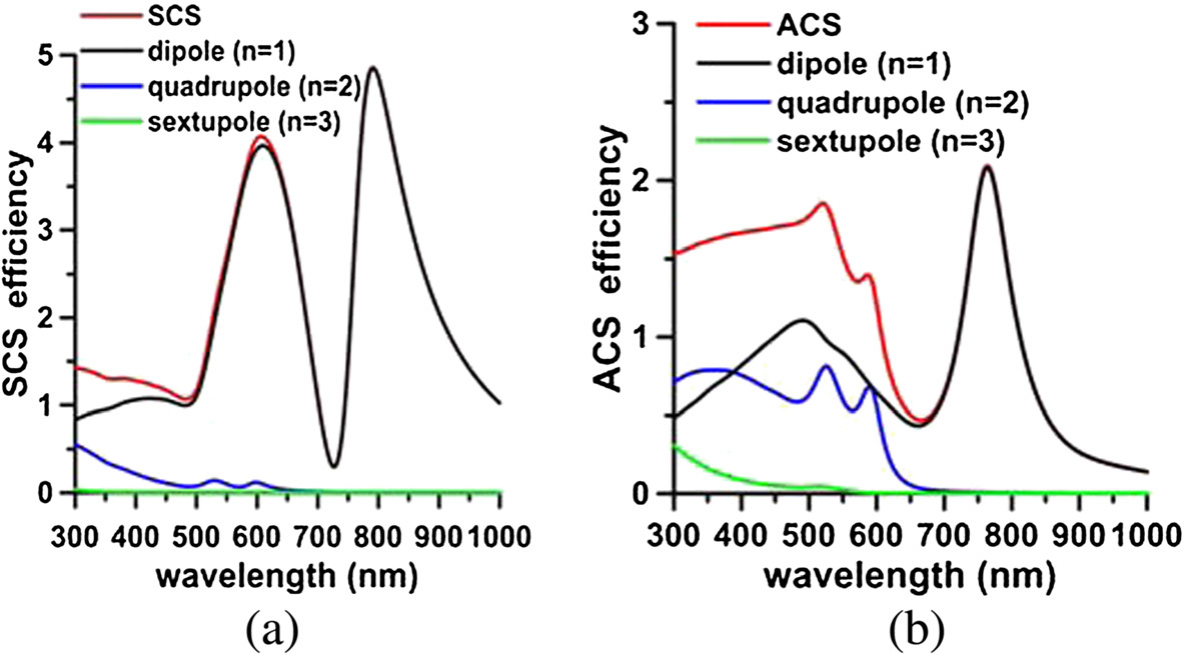
**Scattering efficiencies (a) and absorption efficiencies (b).** Component of each order mode of nanomatryushka. [*a*_*1*_*, a*_*2*_*, a*_*3*_] = [75, 50, 35] nm.

The plasmon modes of a typical nanomatryoshka of size [*a*_1_, *a*_2_, *a*_3_] = [75, 50, 35] nm are analyzed first. The surrounding medium is water. The permittivity of Au is taken from the literature [24]. A radial electric dipole in the proximity of a nanomatryoshka is considered, as shown in Figure 1. Figure 2a,b plots the spectra of the radiative and nonradiative powers, respectively, where *d* = 25 nm. These values are normalized by the radiative power of a free electric dipole in water without a scatterer. Table 1 presents the plasmon modes (dipole and quadrupole modes) and Fano resonances and dips that are obtained from these spectra. The Fano dip divides each of the dipole and quadrupole modes into bonding and anti-bonding modes. In Figure 2, the contributions of each order (*n* = 1, 2, 3,…) of the dyadic Green's functions, which are series solutions in terms of spherical wave vectors, are separated individually from the radiative and nonradiative powers: the dipole mode (*n* = 1), quadrupole mode (*n* = 2), sextupole mode (*n* = 3), octupole mode (*n* = 4), etc. In addition, the scattering cross section (SCS) and absorption cross section (ACS) are calculated using the Mie theory, as presented in Figure 3. The component of each order mode is also separated in Figure 3. These scattering and absorption efficiencies are the normalized SCS and ACS by the cross-sectional area, $a_{1}^{2}$.

**Table 1:** Fano dips and resonances of the dipole and quadrupole modes of nanomatryoshka in water

		**Dipole mode (nm)**			**Quadrupole mode (nm)**		
		**Bonding**	**Fano dip/ resonance**	**Anti-bonding**	**Bonding**	**Fano dip/ resonance**	**Anti-bonding**
I	Dipole						
	*P*_r_	820	740	648	600	568	533
	*P*_nr_		767			590	
	Plane wave						
	SCS	790	727	606	598	571	529
	ACS		765			587	
II	Dipole						
	*P*_r_	850	784	670	616	586	534
	*P*_nr_		810			607	
	Plane wave						
	SCS	830	772	620	614	588	531
	ACS		808			604	

I: [*a*_1_*, a*_2_*, a*_3_] = [75, 50, 35] nm, II: [*a*_1_*, a*_2_*, a*_3_] = [75, 50, 37] nm. *d* **=** 25 nm. Fano dip: *P*_r_ or SCS. Fano resonance: *P*_nr_ or ACS.

### Dipole mode

**Figure 4:**
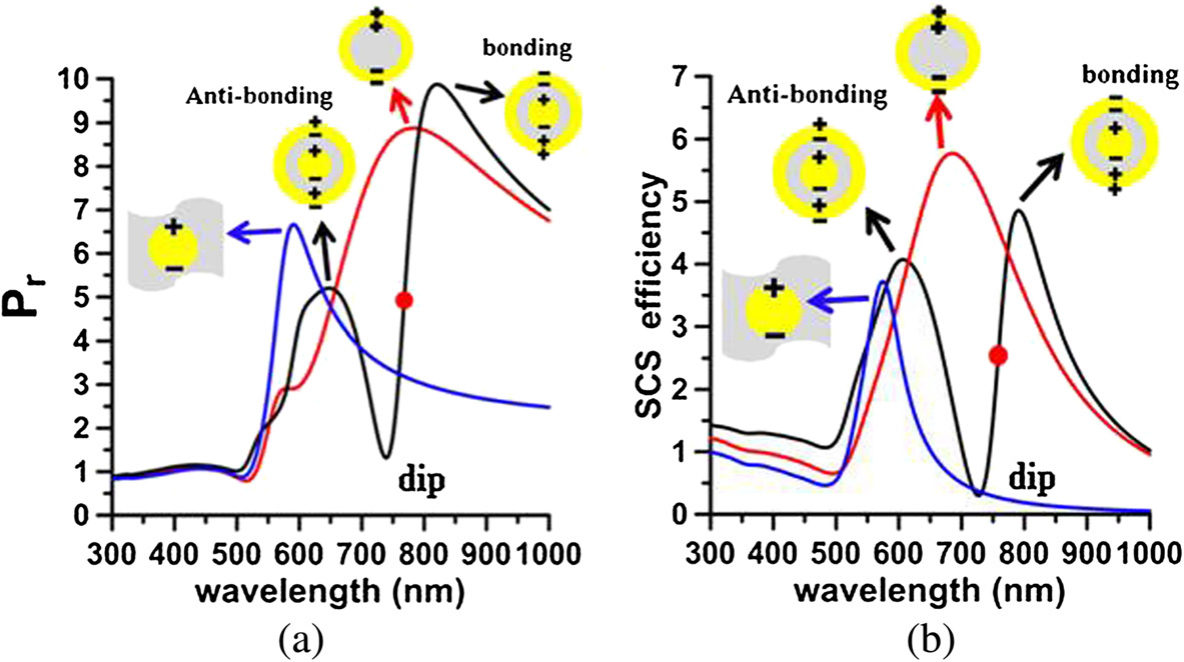
**Radiative powers (*d*** **= 25 nm) (a) and SCS efficiencies (b).** Core in silica (blue curve), nanoshell (red curve), and nanomatryushka (black curve). Red dot: Fano resonance.

Figure 2 shows a pronounced Fano dip in the radiative power (*P*_r_) spectrum at 740 nm and an accompanying peak (Fano resonance) in the nonradiative power (*P*_nr_) spectrum at 767 nm. Similarly, the SCS spectrum from plane wave illumination shows a Fano dip at 727 nm, and an accompanying Fano resonance is observed in the ACS spectrum at 765 nm (Figure 3). The Fano dip is the local minimum of *P*_r_ and SCS, while the Fano resonance is the local peak of *P*_nr_ and ACS; these two are very close to each other. These Fano behaviors are mutually consistent. For comparison, Figure 4a,b presents the corresponding radiative powers and SCS efficiencies of the Au core embedded in silica, nanoshell, and nanomatryoshka, respectively, where *d* = 25 nm. Comparing these spectra with those of a nanomatryoshka reveals that a Fano dip divides the dipole mode of the nanoshell into two parts - the anti-symmetric bonding mode and the symmetric anti-bonding mode. The Fano resonances that are obtained from the *P*_nr_ and the ACS spectra are slightly red shifted from the Fano dips in the *P*_r_ and the SCS spectra [25]. The Fano dip and resonance that are predicted from *P*_r_ and *P*_nr_ are slightly red shifted from those obtained from ACS and SCS.

**Figure 5:**
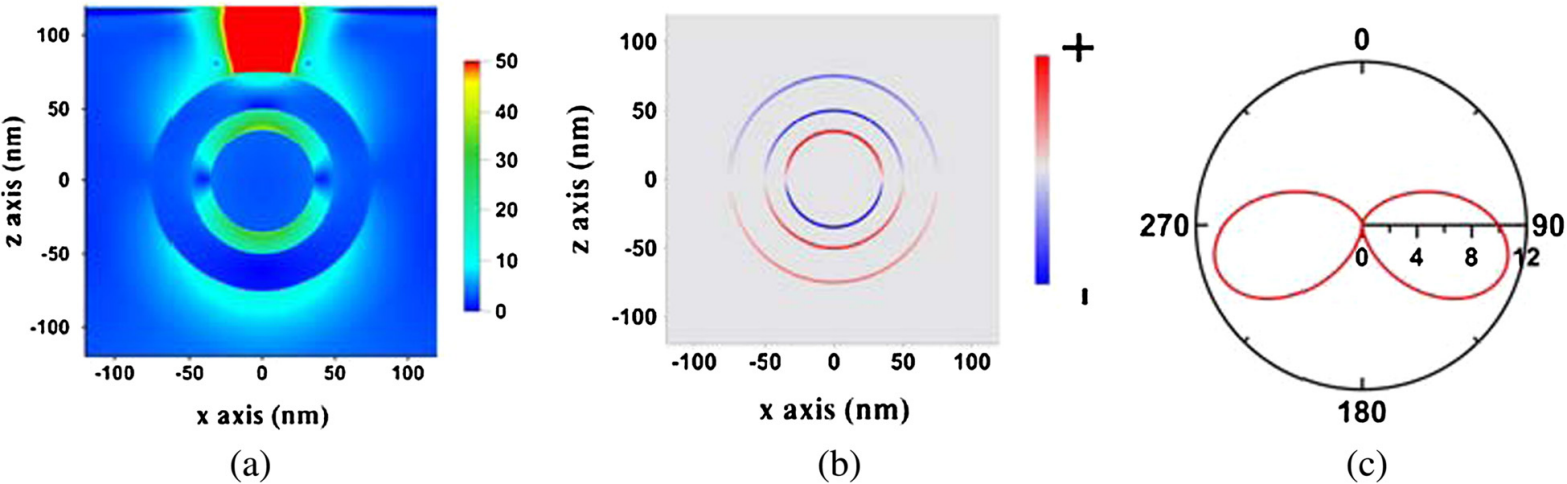
**Electric field (a), surface charge distributions (b), and far-field radiation pattern (c) in *x-z *plane.** Induced by a radial electric dipole interacting with nanomatryushka at the dipolar bonding mode (820 nm), where *d* = 25 nm.

**Figure 6:**
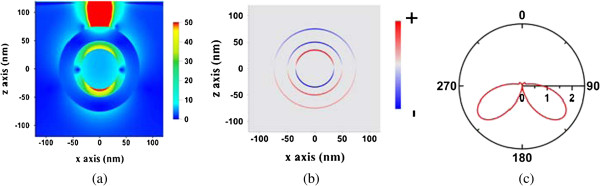
**Electric field (a), surface charge distributions (b), and far-field radiation pattern (c) in *x-z *plane.** Induced by a radial electric dipole interacting with nanomatryushka at the dipolar Fano dip (740 nm), where *d* = 25 nm.

**Figure 7:**
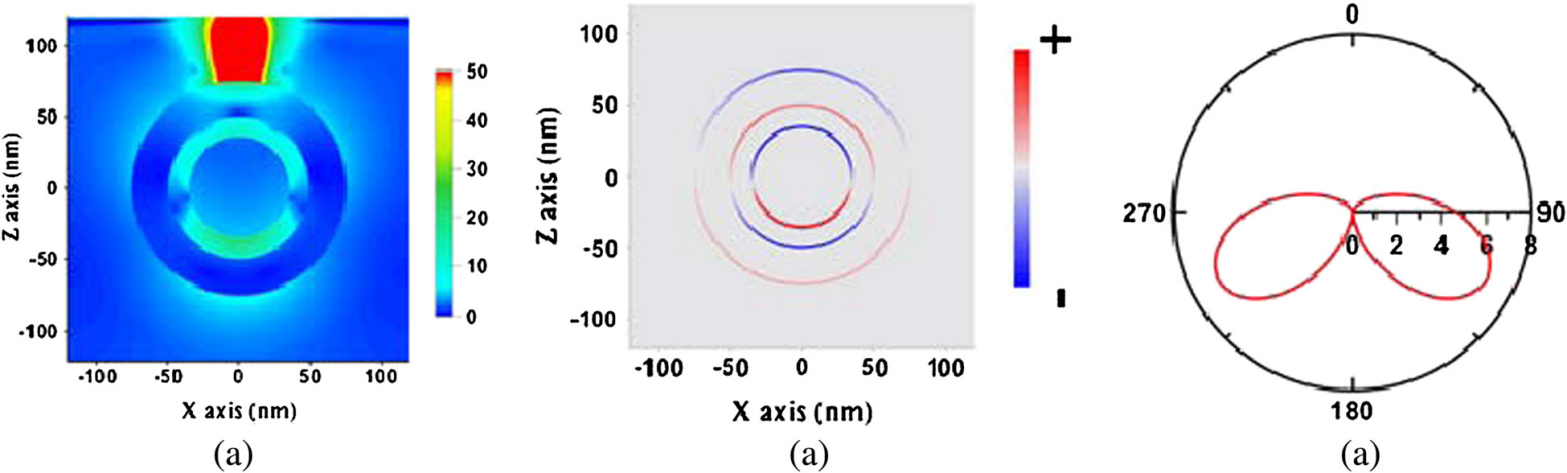
**Electric field (a), surface charge distributions (b), and far-field radiation pattern (c) in *x-z *plane.** Induced by a radial electric dipole interacting with nanomatryushka at the dipolar anti-bonding mode (648 nm), where *d* = 25 nm.

The electric field, surface charge distribution, and far-field radiation pattern in the *x-z* plane of the dipolar bonding mode (820 nm), the Fano dip (740 nm), and the dipolar anti-bonding mode (648 nm) are studied and shown in Figures 5, 6, and 7, respectively. Here, the surface charge we used is defined as Re(*ϵE*_*n*_) on the surface of the Au shell or Au core, which is the real part of the normal displacement field. The red and blue curves represent the positively and negatively charged areas, respectively. The surface charge distributions of both cases are only slightly different. However, a stronger coupling between the Au shell and the Au core occurs in the near field at the Fano dip inducing destructive interference, compared to the field at the dipolar bonding mode. As a result, the strongest internal dissipation in the nanomatryoshka and the least far-field radiation occur at the Fano dip.

**Figure 8:**
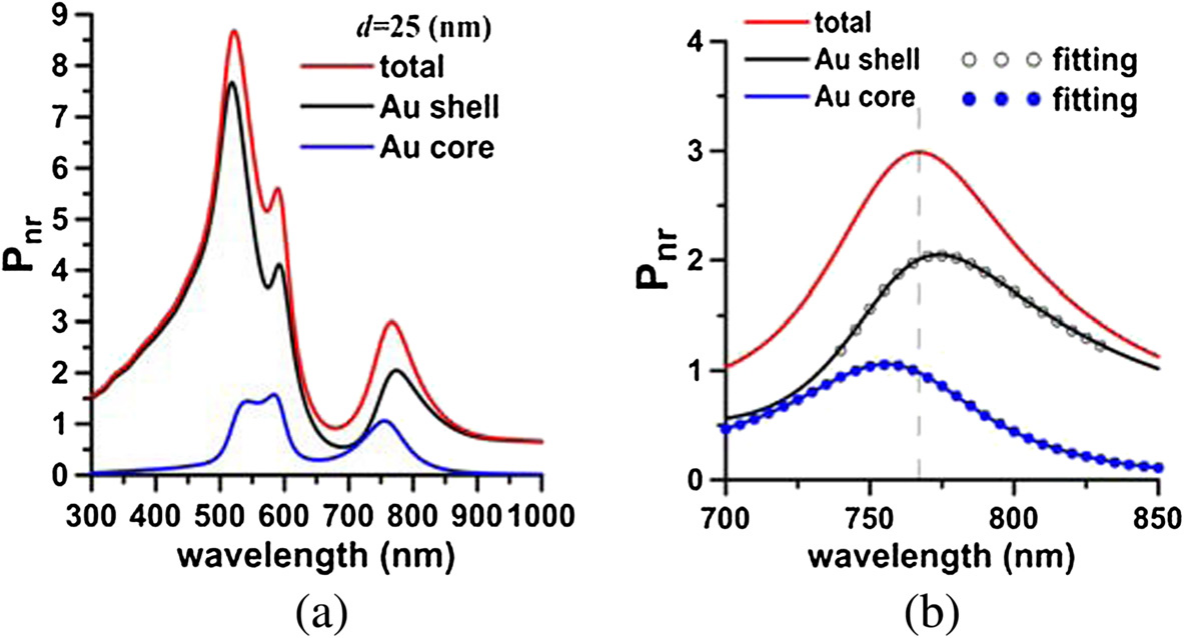
**Nonradiative power and components (a) and fitting Fano line-shape functions (b).** Nonradiative power of nanomatryushka and components of the Au shell and Au core **(a)**. Fitting Fano line-shape functions for Au shell and Au core **(b)**. Fano factors: *q*_1_ = -3.99 (shell) and *q*_2_ = 5.83 (core). *d* = 25 nm.

**Figure 9:**
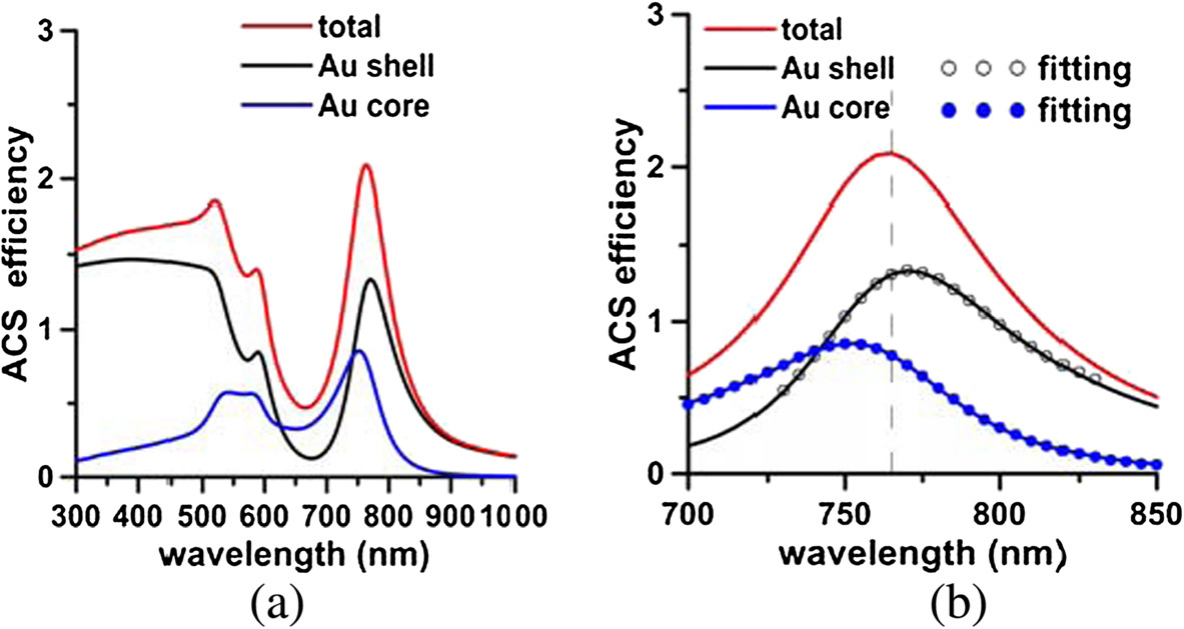
**ACS and components (a) and fitting Fano line-shape functions (b).** ACS of nanomatryushka and components of Au shell and core **(a)**. Fitting Fano line-shape functions for Au shell and core **(b)**. Fano factors: *q*_1_ = -6.19 (shell) and *q*_2_ = 3.95 (core).

The degree of the coupling between the Au shell and the core at the Fano resonance is examined. The nonradiative power spectrum of a nanomatryoshka that is irradiated by an electric dipole is decomposed into two components (for the Au shell and the Au core) according to Equations 2 and 3. Figure 8a plots the $P_{\mathrm{nr}}^{s}$ spectrum of the Au shell and the $P_{\mathrm{nr}}^{s}$ spectrum of the Au core. According to Equation 4, the two spectra are fitted with the Fano line-shape function in the region 700 to 850 nm, as shown in Figure 8b. Table 2 presents the fitting parameters. The Fano factors of the Au shell and the Au core are *q*_1_ = -3.99 and *q*_2_ = 5.83, respectively, for *d* = 25 nm. Similarly, the absorption efficiency of a nanomatryoshka under illumination of a plane wave is also separated into two components associated with the Au shell and the Au core, as presented in Figure 9a. The two absorption spectra are fitted using Equation 4; the Fano factors of the Au shell and the Au cores are *q*_*1*_ = -6.19 and *q*_2_ = 3.95, respectively, as presented in Figure 9b. For the case with a larger distance (*d* = 30 nm), the Fano factors are *q*_1_ = -4.03 and *q*_2_ = 5.79, whose values are in between those of *d* = 25 nm and the plane wave. The latter can be regarded as the responses of *d* → *∞*. According to the analysis herein, these Fano factors of electric dipole irradiation and plane wave illumination are consistent.

**Table 2:** Parameters of Fano line-shape function for Au core and shell of nanomatryoshka at dipole and quadrupole modes

		**Dipole mode**				**Quadrupole mode**			
		***A***	***λ***_**0**_	***δ***_***f***_	***Q***	***A***	***λ***_**0**_	***δ***_***f***_	***Q***
I	Dipole (*d* = 25 nm)								
	Core	0.0302	762.6	42.3	5.83	0.1611	592.2	27.7	2.97
	Shell	0.1208	762.2	46.4	-3.99	0.0301	590.6	23.2	-11.63
	Dipole (*d* = 30 nm)								
	Core	0.0241	762.6	42.3	5.79	0.1265	592.5	28.2	2.87
	Shell	0.0901	762.6	45.2	-4.03	0.0181	591.2	22.8	-12.40
	Plane wave								
	Core	0.0513	762.4	43.7	3.95	0.1239	601.1	43.1	1.89
	Shell	0.0338	763.9	40.8	-6.19	0.0042	589.1	24.6	-14.06
II	Dipole (*d* = 25 nm)								
	Core	0.0287	807.6	34.7	7.17	0.0847	607.3	22.7	4.34
	Shell	0.0683	808.2	38.8	-6.08	0.0209	607.1	22.3	-12.74
	Plane wave								
	Core	0.0451	808.1	35.7	4.64	0.0528	610.7	33.2	2.85
	Shell	0.0191	808.4	33.5	-8.88	0.0031	604.7	24.7	-15.04

I: [*a*_*1*_*, a*_*2*_*, a*_*3*_] = [75, 50, 35] nm, II: [*a*_*1*_*, a*_*2*_*, a*_*3*_] = [75, 50, 37] nm.

**Figure 10:**
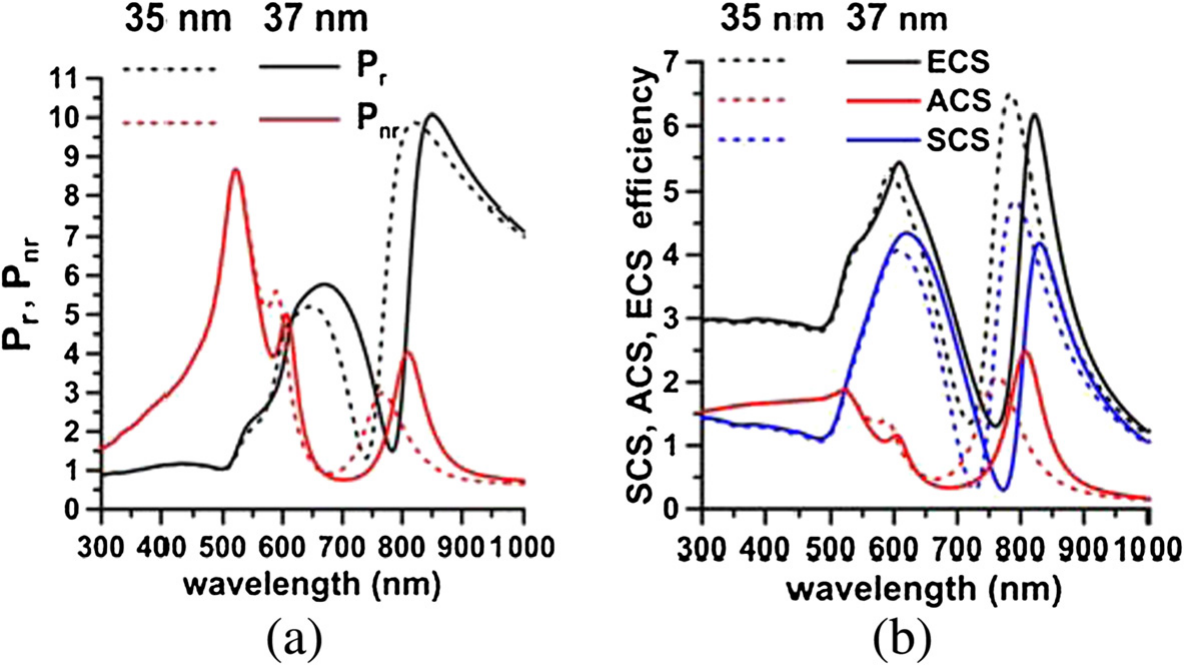
**Radiative and nonradiative powers (a) and SCS and ACS of nanomatryushka (b).** [*a*_1_*, a*_2_*, a*_3_] = [75, 50, 35] nm and [*a*_1_*, a*_2_*, a*_3_] = [75, 50, 37] nm (*d* = 25 nm). ECS = SCS + ACS.

The Fano factors reflect the degree of the internal plasmonic coupling between the Au core and the Au shell. The gap between the Au core and shell is investigated to examine the effect of coupling on the Fano factors. The size of the Au core is increased (say 37 nm) to thin the silica interlayer to increase the internal coupling between the Au core and the Au shell, while keeping the other dimensions of the nanomatryoshka fixed. Figure 10a plots the radiative and nonradiative powers. Figure 10b presents the plane wave responses of SCS and ACS. Figure 10 indicates the red shifts of the plasmon modes (dipole and quadrupole modes) and the Fano dips of [*a*_1_*, a*_2_*, a*_3_] = [75, 50, 37] nm (*t*_2_ = 13 nm) from those of [*a*_1_*, a*_2_*, a*_3_] = [75, 50, 35] nm (*t*_2_ = 15 nm), where *d* = 25 nm. The Fano factors of [*a*_1_*, a*_2_*, a*_3_] = [75, 50, 37] nm from the spectra of the nonradiative power and the absorption efficiency, are *q*_1_ = -6.08, *q*_*2*_ = 7.17 and *q*_1_ = -8.88, *q*_2_ = 4.64, respectively, as listed in Table 2. Comparing these results with those of [*a*_1_*, a*_2_*, a*_3_] = [75, 50, 35] nm, we conclude that the absolute values of Fano factors increase with the internal coupling in the dipole mode.

### Quadrupole mode

For the quadrupole mode, another Fano dip is observed in the radiative power spectrum (*n* = 2) in Figure 2a at 568 nm between the bonding mode and the anti-bonding mode for *d* = 25 nm. The corresponding Fano resonance is observed at 590 nm in the nonradiative power spectrum of Figure 2b. Notice that the peak at 530 nm in Figure 2b is associated with the interband transition (absorption band), rather than any plasmon mode. Accordingly, the absorption band at 520 to 530 nm is observed for each order (*n* = 1, 2, 3,…) component of the nonradiative power. Similarly, the Fano dip at 571 nm in the SCS spectrum (*n* = 2) for a plane wave and the Fano resonance at 587 nm in the ACS spectrum are observed in Figure 3. In contrast to the dipole mode, the quadrupolar bonding and anti-bonding modes and the Fano dip are not pronounced in the radiative power or SCS spectra; only an indication of a shoulder next to the dipolar anti-bonding mode is observed. However, using the order mode analysis, we can identify these features of the quadrupole mode from the component of *n* = 2.

Subsequently, the components of the Au shell and core are decomposed from the nonradiative power spectrum of the nanomatryoshka, and then fitted by the Fano line-shape function in the region of 550 to 650 nm. The Fano factors for *t*_2_ = 15 nm that are extracted from $P_{\mathrm{nr}}^{s}$ and $P_{\mathrm{nr}}^{c}$ are *q*_1_ = -11.63 and *q*_2_ = 2.97, respectively, where *d* = 25 nm. The Fano factors that are obtained from the absorption efficiency spectra of the Au core and the Au shell are *q*_1_ = **-**14.06 and *q*_2_ = 1.89 (Table 2). In contrast, the Fano factors of a nanomatryoshka with a thinner silica layer of *t*_2_ = 13 nm are *q*_1_ = -12.74, *q*_2_ = 4.34 (nonradiative power) and *q*_1_ = -15.04 and *q*_2_ = 2.85 (ACS), respectively. Comparing the results of *t*_2_ = 13 nm and *t*_2_ = 15 nm, we find that stronger internal interferences between two coupled nanostructures (Au shell and core) correspond to larger Fano factors, again. In summary, as the silica layer becomes thinner, the internal coupling between the Au shell and the Au core increases, as revealed by the increase in the Fano factors for both dipole and quadrupole modes.

## Conclusions

The Fano resonances and dips of an Au-SiO_2_-Au nanomatryoshka induced by an electric dipole or a plane wave were investigated theoretically. A Fano dip is the local minimum in the radiative power spectrum (electric dipole) or the scattering efficiency spectrum (plane wave), which is caused by the coupling of destructive interference between the plasmon modes of the Au core and the Au shell. The corresponding Fano resonance is the local maximum of the nonradiative power spectrum (electric dipole) or absorption efficiency spectrum (plane wave), which is very close to the Fano dip. Numerical results herein reveal that a Fano dip divides each of the dipole and the quadrupole modes into bonding and anti-bonding modes. This is to say that the Fano dip (resonance), which is a dark mode, is a phenomenon that arises from the maximum coupling between the Au shell and the core, which induces the strongest internal dissipation and the least radiation. Moreover, the Fano factors of the Au core and the Au shell of a nanomatryoshka quantify coupling around the Fano resonance. These Fano factors that are obtained from the nonradiative power spectrum of an electric dipole are in accordance with those obtained from the absorption spectrum of a plane wave. Additionally, these Fano factors were found to increase with plasmonic coupling.

## Abbreviations

LSPR: Localized surface plasmon resonances; ACS: Absorption cross section; ECS: Extinction cross section; NIR: Near-infrared; QD: Quantum dot; SCS: Scattering cross section.

## Competing interests

The authors declare that they have no competing interests.

## Authors’ contributions

JWL drafted the manuscript. HCC developed the code and calculated the EM field and plotted the figures. MKK derived the equations and developed the code, revised the manuscript, and approved the final version. All authors read and approved the final manuscript.
